# A historical review of multiple system atrophy with a critical appraisal of cellular and animal models

**DOI:** 10.1007/s00702-021-02419-8

**Published:** 2021-10-06

**Authors:** David J. Marmion, Wouter Peelaerts, Jeffrey H. Kordower

**Affiliations:** 1 Parkinson’s Disease Research Unit, Department of Neurobiology, Barrow Neurological Institute, Phoenix, AZ USA; 2 Laboratory for Neurobiology and Gene Therapy, Department of Neurosciences, Leuven Brain Institute, KU Leuven, Leuven, Belgium; 3 ASU-Banner Neurodegenerative Disease Research Center, Biodesign Institute, Arizona State University, Tempe, AZ USA

**Keywords:** Multiple system atrophy, Alpha-synuclein, Oligodendrocytes, Glioneuronal degeneration, Pathology, Animal models

## Abstract

Multiple system atrophy (MSA) is a progressive neurodegenerative disorder characterized by striatonigral degeneration (SND), olivopontocerebellar atrophy (OPCA), and dysautonomia with cerebellar ataxia or parkinsonian motor features. Isolated autonomic dysfunction with predominant genitourinary dysfunction and orthostatic hypotension and REM sleep behavior disorder are common characteristics of a prodromal phase, which may occur years prior to motor-symptom onset. MSA is a unique synucleinopathy, in which alpha-synuclein (aSyn) accumulates and forms insoluble inclusions in the cytoplasm of oligodendrocytes, termed glial cytoplasmic inclusions (GCIs). The origin of, and precise mechanism by which aSyn accumulates in MSA are unknown, and, therefore, disease-modifying therapies to halt or slow the progression of MSA are currently unavailable. For these reasons, much focus in the field is concerned with deciphering the complex neuropathological mechanisms by which MSA begins and progresses through the course of the disease. This review focuses on the history, etiopathogenesis, neuropathology, as well as cell and animal models of MSA.

## Introduction

### Historical review of MSA

Multiple system atrophy (MSA) is a rare, universally fatal, and progressive neurodegenerative disease, clinically characterized by parkinsonian, cerebellar, and dysautonomic features in any combination. The predominant disease feature may evolve as the disease progresses, and which ultimately may or may not encompass all aspects of the disease symptomology. Previously, the disease we now call MSA was separated into three distinct diseases based on symptomatology including Shy–Drager syndrome as well as neuropathology including striatonigral degeneration (SND) and olivopontocerebellar atrophy (OPCA).

The first clinically reported cases of MSA were published in 1900, when Dejerine and Thomas described two cases of adult-onset (42 and 52 years of age) sporadic ataxia, presenting with dysarthria, akinesia, rigidity, and brisk leg reflexes. The patients developed urinary incontinence and probable postural hypotensive symptoms. Both patients died three years following diagnosis (Dejerine 1900; Quinn 1989). Pathological analysis of these cases revealed degeneration in the olives, pons, and cerebellum creating the term *olivopontocerebellar atrophy*. Of note, the striatum and substantia nigra were neither examined nor commented on, so the presence of degeneration in these areas is unknown in these cases.

In 1925, Bradbury and Eggleston published a report of three middle-aged men (36, 47, and 60) who presented with postural hypotension along with anhidrosis and impotence, for the first time suggesting the existence of orthostatic hypotension (OH) of an idiopathic origin (Bradbury and Eggleston 1925). In the following years, reports were made associating autonomic failure with parkinsonian features, such as publications by Langston and Young where middle-aged men (56, 43) had a history of autonomic failure followed by parkinsonism, antecollis, and husky uncontrollable speech (Langston 1936).

In 1960, Shy and Drager saw four cases and reported “a neurological syndrome associated with orthostatic hypotension”, with all cases having marked autonomic dysfunction, slurred speech, impaired coordination, and tremors in the hands at rest. In addition, pyramidal signs and reduced facial expressions were noted. The patients died 5–7 years following disease, and post-mortem evaluation showed cell loss and/or gliosis throughout the brain, but most prominent in the caudate, putamen, globus pallidus, substantia nigra, olives, pons, locus coeruleus, cerebellum, and intermediolateral cell columns of the spinal cord (Shy and Drager 1960).

In 1960 and 1961, van der Eecken, Adams, and van Bogaert published a series of case reports in which they described pronounced shrinkage and discoloration of the putamen and globus pallidus, as well as depigmentation of the substantia nigra, with the striking disappearance of the small cells of the caudate and putamen (Van der Eecken 1960). The authors went on to describe three patients with sporadic disease presenting with a severe akinetic rigid parkinsonian syndrome with brisk reflexes, cerebellar ataxia, and multiple dysautonomic symptoms. Post-mortem examination revealed striatonigral degeneration that was accompanied with cerebellar, olivary and pontine degeneration. One case reported Lewy bodies in the substantia nigra. It was noted by the authors that the findings did not fit with the classical pathological description of Parkinson’s disease (PD) and were indicative of a different pathological entity (Adams et al. 1961). This occurred prior to the levodopa era and thus the degree of clinical response to levodopa was unavailable to differentially diagnose Parkinson’s disease from MSA. Many previous cases of striatal pathology described in earlier times were initially believed to be part of the spectrum of PD. As such, in 1930, Messing described the case of a 62-year-old woman who presented with “the classical picture of PD”, however, upon pathological examination revealed “total atrophy of the ponto-cerebellar system, partial atrophy of the inferior olives and their connections, marked atrophy of the supero-lateral portions of the cerebellum and of the vermis…and fibrosis of the globus pallidus…together with demyelination of the putamen and the anterior portion of the caudate nucleus…This was certainly a case of Parkinson's disease, with an unusual additional lesion: atrophy of the cerebellar pathways" (Messing 1930).

The distinguished movement disorders specialist Professor Niall Quinn, described clinicians over decades approaching MSA like “blindfolded men examining different parts of an elephant and coming away with different impressions of the nature of the beast” (Quinn 1989). It was not until 1969 that Graham and Oppenheimer united these different parts of the “beast”- olivopontocerebellar atrophy, Shy–Drager syndrome, and striatonigral degeneration—as a single disease, stating “there is a group of progressive neurological conditions, most often arising during middle life, with symptoms and signs of lesions affecting several central nervous structures…what is needed is a general term to cover this collection of overlapping progressive pre-senile multi-system degenerations. We wish to avoid the multiplication of names for ‘disease entities’ which in fact are merely the expressions of neuronal atrophy in a variety of overlapping combinations. We, therefore, propose to use the term “Multiple System Atrophy” (Graham and Oppenheimer 1969).

Twenty years after uniting these seemingly different clinical manifestations under one name, the key pathological hallmark of MSA was discovered. In 1989, Papp and Lantos used the Gallyas method of silver impregnation to first describe the accumulation of insoluble protein aggregates in the brains of 11 patients previously diagnosed with either striatonigral degeneration, olivopontocerebellar atrophy, or Shy–Drager syndrome. These aggregates resembled neurofibrillary tangles typical of Alzheimer’s disease; however, histological methods clearly showed these proteinaceous inclusions were located in the cytoplasm of oligodendroglia (Papp et al. 1989). Thus, the inclusions were named glial cytoplasmic inclusions (GCIs) or Papp-Lantos bodies and represent a pathological hallmark specific to MSA. Subsequent studies have shown that the accumulation of GCIs seem to impact the pathology of MSA, as the degree of the burden of GCIs strongly correlate with the level of neuronal loss and demyelination, and that the formation of GCIs occurs prior to the degeneration of axons or neurons (Papp and Lantos 1994; Ozawa et al. 2004). Later, Spillantini and colleagues showed in a seminal paper in 1998 that alpha-synuclein (aSyn), the major component of Lewy bodies (the pathological hallmark of PD) was also the main constituent of insoluble filamentous glial aggregates found in MSA (Spillantini et al. 1998). This finding provided the first ever pathological link between PD, MSA, and dementia with Lewy bodies, uniting these diseases with a common pathological protein as alpha-synucleinopathies.

### Epidemiology, etiology, and genetics

MSA is a rare disease with an estimated incidence between 0.6 and 0.7 cases per 100,000 person-years (Bower et al. 1997; Fanciulli and Wenning 2015); however, studies out of Russia and Sweden have stated incidences of 0.1 and 2.4 cases per 100,000 person-years, respectively (Linder et al. 2010; Winter et al. 2010). The estimated prevalence of MSA ranges from 1.9 to 4.9 per 100,000 (Tison et al. 2000), which increases up to 7.8 for those over the age of 40 (Schrag et al. 1999) and reaching 12 per 100,000 after 70 years of age (Chrysostome et al. 2004).

The distribution of pathology allows for stratification into two MSA subtypes, MSA-P; a parkinsonian variant characterized by nigrostriatal and striatonigral degeneration, and MSA-C; a cerebellar variant characterized by olivopontocerebellar atrophy (Wenning et al. 1997). Despite this stratification, pathology is typically not solely restricted to regions defined by one subtype but exists in combination where one variant displays a predominant pathology. A distribution of MSA phenotype has emerged based on data from large patient registries, highlighting a predominance of MSA-P (70–80% of MSA cases) in North America (May et al. 2007), Europe (Wenning et al. 1994a, b, 1997, 2013), and Korea (Kim et al. 2011), while MSA-C accounts for 67–84% of cases in Japan (Watanabe et al. 2002; Yabe et al. 2006; Ozawa et al. 2010). Despite this distribution in MSA phenotype, average age of onset remains to be similar throughout North America, Europe, Korea, and Japan, with symptoms emerging in the fifth to sixth decade of life (Saito et al. 1994; Bower et al. 1997; Wullner et al. 2007; Kollensperger et al. 2010; Kim et al. 2011). However, instances of young-onset MSA, where disease onset occurs before 40 years of age, and late-onset MSA variants, with disease onset after 75 years old, have been described (Batla et al. 2018, Fanciulli et al. 2019). Although less common, other subtypes of atypical MSA have been described. In these cases, there is restricted and severe neuropathology accompanied by widespread and high GCI burden that extends beyond the affected site. We will focus on these cases of ‘minimal change MSA’ and other atypical forms of MSA in a separate section below.

Unlike PD, MSA seems to affect both sexes equally and progresses more rapidly than PD, with a mean survival of 6–9 years following symptom onset (Quinn 1989; Papapetropoulos et al. 2007; Schrag et al. 2008; Wenning et al. 2008; Low et al. 2015). Of note, however, cases of MSA have been reported with survival up to 20 years, while others are extremely rapidly progressing with disease duration as short as 2 years (Watanabe et al. 2002; Petrovic et al. 2012). The specific factors that dictate the speed of survival and deterioration remain unclear; however, early development of severe autonomic failure has been shown to triple the risk for shorter survival (Watanabe et al. 2002; Tada et al. 2007; O'Sullivan et al. 2008; Figueroa et al. 2014). A prospective, multicenter study showed that patients presenting with pure autonomic failure are at high risk for phenoconverting to manifest CNS synucleinopathy. Specifically, those who phenoconverted to MSA were found to do so faster than LB synucleinopathies, and to have a younger age at onset of autonomic failure, sever bladder/bowel disfunction, preserved olfaction and a cardiac chronotropic response upon tilt > 10 beats per minute (Kaufmann et al. 2017). Additionally, early falls, early development of combined autonomic and motor features, and severe dysautonomia were recently indicated as unfavorable predictors of survival, while neither sex nor MSA phenotypes were predictors of survival (Glasmacher et al. 2017). Moreover, in one meta-analysis, it was shown that patients who had an onset of MSA later in life had a shorter survival time (Ben-Shlomo et al. 1997).

Like PD, ~ 20–75% of MSA cases are characterized as having a prodromal phase occurring months to several years prior to motor presentation. In the prodromal phase, non-motor symptoms include cardiovascular autonomic failure, REM sleep behavior disorder, respiratory disorders, sexual and urogenital dysfunction (Gilman et al. 2008; Jecmenica-Lukic et al. 2012; Xie et al. 2015; Xia and Postuma 2020). Patients often develop urinary symptoms with the appearance of neurological signs. However, it is estimated that over 20% of all MSA patients experience sexual or urological symptoms as their initial complaint (Kirchhof et al. 2003), before the occurrence of orthostatic hypotension, autonomic dysfunction or any other central symptoms. At onset these patients develop urinary urgency and incontinence after which urinary retention with voiding difficulties become increasingly apparent (Panicker et al. 2020). This early lower spinal or infrasacral involvement of the urogenital system has led to the hypothesis that a subtype of MSA might exist and and start within the spinal cord degeneration or impairment of sympathetic or parasympathetic nerves innervating these peripheral sites (Panicker et al. 2020). The prodromal involvement of the urogenital system is also reflected by the unusual high frequency of urinary tract infections that are prominent in MSA. More than half of MSA patients suffer from recurrent or chronic UTIs (Papapetropoulos et al. 2007; Jecmenica-Lukic et al. 2012), both before and after the formal diagnosis of MSA. Peripheral infections, including urinary tract infections, can give rise to sepsis and is a frequent cause of death in MSA (Papatsoris et al. 2008).

The precise cause of MSA remains unknown but is likely to be multifactorial, potentially with a combination of genetic predisposition and environmental factors, although currently MSA is generally considered to be a sporadic disease (Fanciulli and Wenning 2015) and a family history of parkinsonism or cerebellar ataxia is considered as a non-supporting feature in diagnostic criteria (Gilman et al. 2008). However, MSA pedigrees with both autosomal dominant and autosomal recessive inheritance patterns have been reported in Europe and Asia (Wullner et al. 2004; Hara et al. 2007; Vidal et al. 2010; Hohler and Singh 2012; Fujioka et al. 2014; Itoh et al. 2014). The prominent difference between the presentation of MSA-P and MSA-C variants across different ethnicities suggests a genetic predisposition towards a specific variant of MSA (Ozawa et al. 2012). A loss-of-function mutation in the gene *COQ2*, which encodes for the coenzyme Q10 (COQ10), has been reported in Japanese familial and sporadic MSA cases, as well as other East Asian countries (Multiple-System Atrophy Research Collaboration 2013; Ogaki et al. 2014; Quinzii et al. 2014; Lin et al. 2015; Zhao et al. 2016). This link between V393A mutations in the *COQ2* gene and MSA patients has not been confirmed in European, North American, and Korean populations (Jeon et al. 2014; Schottlaender et al. 2014; Sharma et al. 2014; Ronchi et al. 2016; Sailer et al. 2016). Moreover, decreased levels of COQ10 have been reported in plasma and the cerebellum of MSA-C patients, thus mutations in *COQ2* and altered levels of COQ10 may be region- or subtype-specific and may not represent common genetic factors for MSA (Barca et al. 2016; Schottlaender et al. 2016). Decreased levels of COQ10 may contribute to MSA pathogenesis due to decreased electron transport in the mitochondria and increased vulnerability to oxidative stress (Nakamoto et al. 2018).

Duplications and triplications of the *SNCA* gene, which codes for aSyn, may cause familial PD with glial cytoplasmic inclusions in some members (Gwinn et al. 2011). An A53E SNCA mutation was reported in a Finnish patient displaying neuronal and glial aSyn pathology throughout the brain and spinal cord (Pasanen et al. 2014). Additionally, a G51D SNCA mutation was reported in a British pedigree with autosomal dominant juvenile Parkinsonism, which also displayed neuropathological hallmarks of MSA with GCIs but without the typical clinical features of MSA (Kiely et al. 2013, 2015). It is important to note that in PD, greater disease severity and duration leads to increased accumulation of aSyn in oligodendroglia, thus these point mutations may be causing a more aggressive form of PD and not pure MSA (Halliday et al. 2011; Katzeff et al. 2019). Two single-nucleotide polymorphisms of the SNCA locus have been identified in two separate studies (Al-Chalabi et al. 2009; Scholz et al. 2009), however these were not replicated in the largest MSA genome-wide association study (Sailer et al. 2016). This same study by Sailer and colleagues did, however, identify single-nucleotide polymorphisms in other genes, such as MAPT, FBX047, EL0VL7, and EDN1, which may have implications in MSA pathogenesis (Sailer et al. 2016). Other studies have proposed genetic variants for increased MSA susceptibility, such as GBA (Mitsui et al. 2015), SLC1A4, SQSTM1, and EIF4EBP1 (Soma et al. 2008), and LRRK2 (Heckman et al. 2014; Lee et al. 2018; Riboldi et al. 2019), Some of these risk variants were also found in multiplex families where MSA coincided with PD and suggests some overlap in genetic risk between diseases (Mitsui et al. 2015).

No environmental factors have yet to be unequivocally linked to an increased risk of MSA. Initial case-controlled reports in North America and Europe showed an increased risk of developing MSA for those who worked in agriculture, had exposure to plastic monomers and additives, metals and organic solvents (Nee et al. 1991; Vanacore et al. 2005), however, these findings were unable to be replicated in follow-up studies (Cho et al. 2008; Vidal et al. 2008). As observed in PD, two studies have shown a protective effect of smoking in which individuals who smoke tobacco are less likely to get MSA than those who do not (Vanacore et al. 2000; Chrysostome et al. 2004). Interestingly, this protective effect of smoking is not seen in progressive supranuclear palsy, a tauopathy, which may indicate an interaction between smoking and aSyn.

Given the conflicting data surrounding the influence of genetics and the environmental impact on MSA, there is little scientific evidence to correlate these factors with an increased risk of MSA. A recent study identified intestinal inflammation in cases with inflammatory bowel disease as a potential risk factor of MSA using clinical data available from to the Danish National Patient Register (Villumsen et al. 2019). The overall incidence of MSA cases in this study was low which is typical of epidemiological. A definite diagnosis of MSA requires post-mortem evaluation, which is lacking in many of the epidemiological and genetic studies. Larger numbers of pathologically confirmed MSA cases and healthy controls are required in future studies to reach sufficient power (Vanacore 2005; Jellinger 2018).

### Neuropathology

The precise mechanism by which degeneration occurs in MSA remains unclear. Converging evidence from post-mortem and experimental studies suggests that MSA is a primary oligodendrogliopathy where disruptions in the oligo-myelin-axon complex lead to secondary neurodegeneration (Wenning et al. 2008; Jellinger 2015, 2018), rather than MSA being a primary neuronal disorder with secondary GCI accumulation of aSyn from a neuronal origin (Ubhi et al. 2011). However, the presence of aSyn mRNA within oligodendroglia remains controversial and is an important point that to this day requires clear resolution. Initial reports indicating the lack of aSyn mRNA in oligodendroglia have been challenged by recent studies demonstrating the presence of aSyn mRNA in oligodendrocytes from post-mortem MSA tissue (Miller et al. 2005; Jin et al. 2008; Asi et al. 2014). Developmental studies utilizing embryonic stem cells and induced pluripotent stem cells have shown aSyn protein and mRNA in oligodendrocyte precursor cells (OPCs) that diminishes as oligos mature (Djelloul et al. 2015), and experimentally induced expression of aSyn in OPCs hinders development of oligodendroglia and their ability to myelinate (Ettle et al. 2014; May et al. 2014). In related demyelinating disorders, sparse clusters of oligodendrocytes of the spinal cord and the cerebrum express aSyn (Falcao et al. 2018; Jakel et al. 2019). It has been speculated that an overlap between different demyelinating diseases, such as multiple sclerosis (MS) and MSA, might exist (Jellinger and Wenning 2016). Curiously, these aSyn-positive oligodendrocytes in MS cases were mainly found within active or chronic white matter lesions (Lu et al. 2009), suggesting that aSyn expression could be triggered, possibly transiently in demyelinating lesions.

These recent findings suggest that oligodendroglia can express aSyn and that they might be the primary source of aSyn found in MSA GCI’s. However, this does not rule out other potential mechanisms such as passive transmembrane diffusion and endocytosis. The impact of the neuronal endosomal–lysosomal system in the processing of aSyn in PD is well established, while lysosomes contribute to the pathogenesis of MSA, enabling oligodendroglial and neuronal uptake of aSyn (Puska et al. 2018). Reduced oligodendrocyte-derived enriched microvesicles (OEMVs) could be an important mechanism related to pathological aSyn aggregation in oligodendrocytes (Yu et al. 2020) and more recently, aSyn was also isolated from both neuronal- and oligodendroglial-derived exosomes from the blood of MSA patients where it was found to be significantly elevated within these exosomes. Compared to PD exosomes, MSA exosomes contained significantly higher levels of aSyn making blood exosomes potentially useful for biomarkers of MSA (Dutta et al. 2021). In vitro and in vivo studies have experimentally demonstrated the transfer of aSyn from neurons to oligodendrocytes (Kisos et al. 2012; Rockenstein et al. 2012; Reyes et al. 2014; Kaji et al. 2018). Aggregated aSyn can act in a prion-like manner that propagates throughout the neuraxis in MSA (Watts et al. 2013; Peelaerts et al. 2015; Prusiner et al. 2015; Peng et al. 2018; Woerman et al. 2015, 2018, 2019). Supporting the notion that MSA is a primary oligodendrogliopathy, GCIs are first observed in regions where significant neuronal loss occurs and GCI density correlates with the degree of neuron loss (Papp and Lantos 1994; Ozawa et al. 2004; Yoshida 2007). Apart from aSyn pathology in oligodendrocytes, abnormal accumulation of fibrillary aSyn is also present in neuronal cytoplasm inclusions (NCIs) and neuronal nuclear inclusions (NNIs) as well as in neurites human MSA brains examined at post-mortem (Papp and Lantos 1994; Yoshida 2007). Indeed, post-mortem studies of MSA brains have suggested that NNIs develop early in the disease process in the pontine nuclei and inferior olives (Nishie et al. 2004; Wakabayashi et al. 2005; Wakabayashi and Takahashi 2006). NCIs are widely distributed throughout the brain; however, their frequency does not seem to relate to the degree of neuron loss (Ozawa et al. 2004). Neuronal accumulation of aSyn and potential relevance to disease symptomatology and progression are described below in the section entitled “Atypical MSA”. Furthermore, GCIs are the hallmark of MSA and are not seen as frequently in PD, even though both diseases share similar lesion patterns in many overlapping circuits (Halliday 2015).

The accumulation of aSyn causes dysfunction in normal oligodendrocyte, leading to demyelination, axonal damage, reduced neurotrophic support, and subsequent neurodegeneration (Matsuo et al. 1998; Ubhi et al. 2010; Jellinger 2014). The immune system has been shown drive pathogenesis in MSA, as evidence from post-mortem and experimental studies have shown astrogliosis, microglial activation, and recently an infiltration of T cells (Stefanova et al. 2007; Rydbirk et al. 2017; Refolo et al. 2018; Williams et al. 2020). Severe neuron loss is seen in several brain regions, such as the putamen, substantia nigra, inferior olives, pons, and cerebellum. Despite the accumulation of aSyn in oligodendroglia, demyelination, and the proposed pathogenic role of GCIs, the number of oligodendrocytes in MSA brains seems to be preserved or only a modest reduction when compared to healthy controls (Nykjaer et al. 2017; Salvesen et al. 2017).

On the cellular level, oligodendroglial pathology in MSA results in accumulation of another protein, p25α, in oligodendrocytes from patients with MSA (Kovacs et al. 2004; Orosz et al. 2004). P25α, also known as tubulin polymerization-promoting protein (TPPP) (Takahashi et al. 1991), is an oligodendroglial-specific phosphoprotein functionally involved in myelination and stabilization of microtubules (Ovadi and Orosz 2009). Under physiological conditions, p25α resides in the myelin sheath; however, in MSA patients, it is shifted into oligodendroglial cell bodies preceding aSyn aggregation (Song et al. 2007). Furthermore, p25α stimulates aSyn aggregation in vitro (Lindersson et al. 2005), and up to 50% of oligodendroglia show abnormal accumulation of p25α, which is often co-localized with insoluble aggregates of aSyn in GCIs (Song et al. 2007; Wenning et al. 2008). Paralleling the relocation of p25α, a dramatic decrease of total levels of myelin basic protein (MBP) and concurrent increase in degraded MBP is observed. The co-expression of p25α and aSyn in rat oligodendroglial cell line OLN-93 resulted in the formation of pS129 + aSyn inclusions and showed a rapid retraction of microtubules from cellular processes followed by a protracted development of apoptosis (Kragh et al. 2009).

As mentioned previously, aSyn was discovered to be the main component of Lewy bodies in PD, and subsequently identified as a major protein aggregated in GCIs found in patients with MSA, thus linking the process of aggregation of aSyn to the pathogenesis of these diseases (Spillantini et al. 1998). aSyn found in GCIs undergoes a number of post-translational modifications, such as oxidative modifications, tyrosine nitration, and phosphorylation at serine 129 (Duda et al. 2000a, b; Giasson et al. 2000; Kahle et al. 2002; Beyer 2006; Beyer and Ariza 2007). Recent efforts have sought to investigate how the same protein, aSyn, can misfold and accumulate specifically in different cell types in the brain causing distinct diseases. The generation of pure fibrillar aSyn polymorphs with structural and phenotypic differences has led to the belief that different “species” or “strains” of aSyn may be responsible for the heterogeneity of synucleinopathies (Bousset et al. 2013). Supporting this hypothesis, distinct aSyn assemblies, “fibrils” and “ribbons”, displayed vast differences in terms of aSyn pathology and spreading when injected into animals (Peelaerts et al. 2015). Moreover, aSyn extracted from brain samples of PD, DLB, and MSA subjects have shown distinct properties experimentally (Masuda-Suzukake et al. 2013; Watts et al. 2013; Recasens et al. 2014; Peng et al. 2018; Van der Perren et al. 2020). Experiments showing that oligodendrocytes, but not neurons, transform misfolded aSyn into a “GCI strain” have highlighted the importance of the intercellular milieu on generating distinct aSyn strains (Peng et al. 2018). Moreover, GCI-derived aSyn has been shown to be more potent in producing neurodegeneration, aSyn propagating pathology, and inflammation than both PD- and DLB-derived aSyn (Prusiner et al. 2015; Peng et al. 2018; Van der Perren et al. 2020).

The molecular origin of these aSyn MSA protein strains likely depend on the host cellular environment or the interactions between oligodendroglia and aSyn. Unique oligodendroglial factors that seed and interact with aSyn, such as the aforementioned p25α protein can influence the folding landscape of aSyn (Peng et al. 2018; Ferreira et al. 2021). In vitro aggregation of aSyn with oligodendroglial extracts or p25α results in the assembly of an aggressive aSyn strain that causes exacerbated neurotoxicity when injected in mouse brain (Ferreira et al. 2021). Cryo-EM studies have confirmed that MSA fibrils are unique, and that they are structurally distinct compared to fibrils isolated from the brain of people with PD or DLB. Within the core of the MSA fibril, there is a large cavity with protruding charged side chains that are covered by a non-proteinaceous residue. This density could be an important cofactor required for screening these charged residues and the assembly of the MSA strain (Schweighauser et al. 2020). Within different regions of MSA brain, slight variations within the fibril conformation are observed, further indicating that a cloud of related assemblies could exist for a given disease. While an exciting paradigm shift in understanding synucleinopathies, the strain differences need to be more closely examined and reproduced as in vitro amplification of prion-like proteins do not always recapitulate how they behave in vivo.

### Atypical MSA

While MSA-P and MSA-C, in combination with autonomic dysfunction represent the typical MSA presentations, uncommon clinicopathologic forms of MSA exist, in which several subtypes do not fit the current classification (Watanabe et al. 2016). These forms of MSA include minimal change MSA, non-motor MSA and incidental MSA. All present with variable aSyn, glial or neuronal pathology.

The term “minimal change” MSA describes a pathological variant of MSA that is characterized by widespread and typical distribution of GCIs; however, neuronal loss is restricted to the substantia nigra and locus coeruleus (Wenning et al. 1994a, b). Ling and colleagues report that, of the six “minimal change” MSA cases evaluated, three presented with Parkinsonism and three presented with autonomic features (Ling et al. 2015). When compared to MSA controls, subjects with “minimal change” MSA were found to have widespread GCIs with a significant increase of NCIs in the caudate and SN. Moreover, cases of “minimal change” MSA had an earlier age of onset (Mean 38 vs 57.6), a more rapid clinical progression, and a shorter disease duration (mean 5.3 vs 8 years), suggestive of a more aggressive variant of MSA (Ling et al. 2015).

Additionally, Wakabayashi and colleagues reported a case of early “minimal change” MSA of the cerebellar variant. The authors report a 57-year-old-woman with a 15-month disease duration and neuronal loss restricted to olivopontocerebellar system. Despite widespread GCIs throughout the CNS, neuronal aSyn pathology was confined to the pontine and inferior olivary nuclei (Wakabayashi et al. 2005). The authors state that GCI formation is the earliest stage of the degenerative process, which causes destruction of myelin, together with neuronal aSyn accumulation, leading to secondary neuronal degeneration.

A second interesting and rare manifestation of MSA is described as non-motor MSA, which presents clinically with autonomic failure in the absence of motor signs or symptoms (Gaig et al. 2008; Riku et al. 2017). Gaig and colleagues report the case of a 60-year-old man with a history of stridor, RBD and urogenital autonomic disturbances, but never progressed to the development of overt Parkinsonism or cerebellar signs. Neuropathological assessment revealed widespread aSyn-positive GCIs accompanied by severe neuronal loss and gliosis in the cerebellar, brainstem and spinal cord areas with only mild pathology in the putamen, SN, and globus pallidus (Gaig et al. 2008). Riku et al. describe four patients identified as having non-motor MSA, in which symptoms presented as urinary disorders, and progressed to nocturnal stridor, anhidrosis, and orthostatic hypotension. Pathologically, marked neuronal loss and gliosis was observed in the intermediolateral column and Onuf’s nucleus, as well as severe loss of serotonergic neurons in the ventrolateral medulla and the nucleus raphe obscurus, with abundant GCIs in the medullary reticular formation, which drastically differs from classical MSA cases (Riku et al. 2017). In these described reports of non-motor MSA, all subjects suffered from inspiratory stridor, depletion of medullary serotonergic neurons, and died of sudden death, and it has been postulated that the loss of serotonergic neurons could be responsible for the sudden death observed in MSA (Tada et al. 2009).

Aoki and colleagues describe another atypical MSA subtype, in which patients present clinically with frontotemporal dementia symptoms with the addition of widespread GCI pathology (Aoki et al. 2015). None of the patients displayed autonomic dysfunction; however, all had severe limbic and cortical aSyn pathology as both GCI and NCI formations. Neuronal inclusions were heterogeneous in nature but included aSyn positive Tau negative Pick body-like inclusions that strongly associated with neuronal loss in the hippocampus and amygdala.

Only in rare instances, GCIs are found in autopsy cases of neurologically normal individuals. However, there are reports of cases with GCI pathology in the SN accompanying neuronal loss but without demyelination (Fujishiro et al. 2008). The two cases reported by Fujishiro and colleagues were 82 and 96 years of age, and as such one may consider the observed GCI pathology an age-related phenomenon, classifying them as “preclinical/prodromal” or “incidental MSA”.

### MSA Models

One reason MSA remains an elusive and puzzling disease to study is the paucity of reliable animal models. This is due, in part, to the complexity of neuropathological features and a questionable genetic influence. Over the years, great effort has gone into the production of in vitro and in vivo MSA models, and recent technological advancements have allowed for the development of more accurate models.

Initially, in vitro modeling of MSA relied on aSyn expression in an astrocytoma cell line U373 (Stefanova et al. 2001, 2002; Stefanova et al. 2003a, b), primary mix rat glial cultures (Stefanova et al. 2002), primary oligodendroglial cell line OLN-93 (Kragh et al. 2009), and primary rodent oligodendroglial cell line CG4 (May et al. 2014; Valera et al. 2017). Using vectors to overexpress full-length human wild-type aSyn (1–140) or C-terminally truncated aSyn (1–111) in astrocytoma cell line U373 and mixed primary mixed glial cultures, Stefanova and colleagues demonstrated widespread fibrillar aSyn aggregates, which were more abundant in C-terminally truncated aSyn-expressing cells. aSyn overexpression in these cells caused neuronal death and increased susceptibility to oxidative stress. These observations were observed in both astrocytes and oligodendrocytes overexpressing aSyn (Stefanova et al. 2001, 2002; 2003a, b). To investigate the potential of microtubule stabilizing protein p25α to stimulate aSyn aggregation, Kragh and colleagues co-expressed human p25α and aSyn in the rat oligodendroglial cell line OLN-96. A rapid relocation of microtubules from the cellular processes to the perinuclear region was observed. This was followed by lasting apoptosis with caspase-3 activation and nuclear chromatin condensation. This response was dependent on the phosphorylation of aSyn at serine 129, and the co-expression of p25α and aSyn was required, as neither protein alone elicited these toxic effects (Kragh et al. 2009). Subsequent studies showed that the overexpression of p25α or aSyn accelerated the recruitment of endogenous aSyn following the addition of aSyn preformed fibrils (PFFs) (Mavroeidi et al. 2019). These studies highlight the role of p25α in the formation of pathological aSyn species in oligodendroglia and the corresponding pathology in MSA.

May and colleagues used the GC4 oligodendroglial cell line and demonstrated that overexpression of aSyn impairs the maturation of oligodendrocyte progenitor cells (OPCs). Cells expressing aSyn displayed abnormal branching, reduced intracellular MBP, and the inability to properly form myelin sheaths (May et al. 2014). These results may provide insight into the progression of MSA, as dysfunctional aSyn expressing OPCs could be unable to remyelinate damaged areas in the MSA brain.

While these initial in vitro studies have provided valuable insight into mechanistic components of the disease process, the use of cell lines have clear limitations. Various genetic modifications have been performed to immortalize cell lines and drive continuous growth. As such, it may be difficult to distinguish artifact from disease-specific pathology and findings must be validated in other models.

### Stem cell based models of MSA

The advent of cellular reprograming of fibroblasts into induced pluripotent stem cells (iPSCs) has allowed for the study of human neural tissue, and more importantly, patient-derived neural tissue (Takahashi et al. 2007). Utilizing this technology, Djelloul and colleagues successfully reprogramed fibroblasts from patients with MSA, PD, and control subjects into functional oligodendrocytes. By recapitulating human development in a dish, they observed the presence of SNCA transcripts and aSyn protein in oligodendrocyte progenitors, which decreased after maturation into oligodendrocytes (Djelloul et al. 2015). Notably, the transient expression of aSyn was observed in cells derived from MSA and control subjects; however, it does confirm the presence of aSyn mRNA in oligodendrocytes and provides the potential hypothesis that aberrant re-expression of SNCA could be triggered during disease state and provide the source of oligodendroglial aSyn in MSA.

iPSC-derived neurons from MSA and control subjects have been differentiated into dopaminergic and cortical neurons to further investigate mitochondrial function in MSA (Monzio Compagnoni et al. 2018; Nakamoto et al. 2018). To look more closely at the implications of COQ2 mutations on mitochondrial function, Nakamoto and colleagues reprogrammed peripheral blood mononuclear cells from patient with a compound heterozygous COQ2 mutation, an idiopathic MSA patient, and three control lines of diverse descent (Caucasian, African, and Japanese origin). COQ2-mutated MSA derived neurons displayed a significant reduction in the mean area of the inner mitochondrial membrane, as well as reductions in COQ10 and vitamin E levels, and changes in mitochondrial respiratory chain activity, compared to sporadic MSA and control derived neurons (Nakamoto et al. 2018). Monzio Compagnoni and colleagues examined mitochondrial and autophagic dysfunction in iPSC-derived dopamine neurons from MSA patients and healthy controls. This study also found dysregulation involving respiratory chain activity, mitochondrial content, and COQ10 biosynthesis in MSA patient-derived neurons. Additionally, treatment with bafilomycin A, which inhibits the fusion of the autophagosome and the lysosome, yielded increased basal levels of LC3-II in MSA samples indicating impairment in autophagic flux (Monzio Compagnoni et al. 2018). The authors hypothesized a vicious cycle in which mitochondrial dysfunction could be triggered by inadequate autophagy, resulting in impaired mitophagy and the accumulation of dysfunctional mitochondria and further dysregulation of autophagy.

While cell-based models of MSA offer the ability to study the disease in human/patient-derived cells, the fact that MSA affects multiple cell lineages and the difficulty to recapitulate the complex network of the CNS causes limitations to elucidate the progression of pathology and adequately assess the efficacy of novel therapeutics.

### Toxin based in vivo models of MSA

Neurotoxin-based animal models of MSA were initially developed to reproduce the anatomical lesion of striatonigral degeneration underlying the L-DOPA unresponsive Parkinsonism observed in MSA. This effort combined previously established neurotoxin lesion paradigms used to model PD and Huntington’s disease, using 6-hydroxydopamine (6-OHDA) and quinolic acid (QA) to selectively target nigral dopamine neurons and striatal GABAergic medium spiny neurons, respectively. The initial “double toxin-double lesion” model was established by a unilateral injection of 6-OHDA in the median forebrain bundle (MFB) followed by QA in the striatum, which induced a near complete loss of nigral DAergic neurons and a dose-dependent excitotoxic loss of striatal medium spiny neurons. Behaviorally, this model exhibited classical ipsilateral amphetamine induced rotations, which were abolished by fetal allografts (Wenning et al. 1996). A subsequent study investigated the effects of lesion sequence (nigrostriatal degeneration vs striatonigral degeneration) and found that while prior striatal lesioning with QA did not affect 6-OHDA-induced nigral degeneration, initial striatal DAergic denervation from 6-OHDA lesions was found to decrease the neurotoxic effects of QA. A bilateral deficit in the stepping test, as well as an absent levodopa response, was also observed in this double-toxin model (Scherfler et al. 2000; Stefanova et al. 2004; Kollensperger et al. 2007). Ghorayeb and colleagues modified the “double toxin-double lesion” approach to model early-stage MSA-P using simultaneous low-dose QA and 6-OHDA in the lateral striatum. This strategy resulted in exacerbation of QA-induced striatal degeneration, with a slight reduction in nigral neuron degeneration. Behaviorally, deficits in the stepping test were observed and rotational asymmetry was abolished, indicating a dopamine unresponsive phenotype (Ghorayeb et al. 2001).

In an effort to prevent interactions between both sites of degeneration, a “single toxin-double lesion” approach was developed in rats using either 3-nitropropionic acid (3-NP), a succinate dehydrogenase inhibitor, or 1-methyl-4-phenylpyridinium ion (MPP^+^), a mitochondrial complex I inhibitor. Either neurotoxin induces combined degeneration of both striatal and nigral neurons when injected into the striatum, as well as bilateral behavioral deficits (Waldner et al. 2001; Ghorayeb et al. 2002a, b).

Systemic administration of neurotoxins avoids intracerebral injections and produces a progressive neurodegeneration, which can mimic the temporal dimension of the human disease. Different injection paradigms were utilized to explore if the order of neurotoxin administration had any effect on degeneration, which was observed in the “dual-toxin dual-lesion” intracerebral approach. In all administration paradigms, intraperitoneal injection of 1-methyl-4phenyl-1,2,3,6-tetrahydropyridine (MPTP) and 3-NP in mice resulted in the development of bilateral SND (Stefanova et al. 2003a, b; Fernagut et al. 2004). As in the intracerebral dual toxin models, prior administration of MPTP reduced the vulnerability of striatal neurons to 3-NP, and administration of 3-NP first reduced the vulnerability of nigral dopaminergic neurons to MPTP. Locomotor deficits were more pronounced with the initial administration of 3-NP, which correlated with the loss of striatal neurons (Stefanova et al. 2003a, b). Unlike the sequential administration of MPTP and 3-NP, the combined systemic administration of these neurotoxins resulted in a more pronounced neurodegeneration than either toxin alone, and elicited decreased motor performance, altered gait patterns, and impaired balance (Fernagut et al. 2004). This systemic approach was also utilized in nonhuman primates, whereby chronic administration of 3-NP induced a progressive aggravation of parkinsonian motor features in MPTP-treated monkeys, which did not respond to levodopa administration. 3-NP treatment induced hindlimb dystonia that correlated to bilateral striatal degeneration visualized by in vivo MRI (Ghorayeb et al. 2000; Ghorayeb et al. 2002a, b). Intracerebral and systemic toxin-based models were able to reproduce levodopa-unresponsive striatonigral degeneration that is observed in MSA. However, these models were unable to recapitulate any aspect of aSyn + GCI formation or oligodendroglial dysfunction, which are key pathological hallmarks of MSA and important disease features as growing evidence points to MSA being a primary oligodendrogliopathy (Wenning et al. 2008) and for the most part have been discarded.

### Transgenic in vivo models of MSA

To address the concerns that arose regarding neurotoxin-based models of MSA, several transgenic (tg) mouse models have been developed, in which human wild-type aSyn was expressed specifically in oligodendrocytes using different oligodendroglial-specific promoters. Targeted aSyn expression was achieved using the proteolipid (PLP) promoter (Kahle et al. 2002), the myelin basic protein (MBP) promoter (Shults et al. 2005) or the 2′,3′-cyclic nucleotide 3′-phosphodiesterase (CNP) promoter (Yazawa et al. 2005). All three tg lines recapitulate the oligodendroglial accumulation of insoluble alpha-synuclein.

When expression of αSyn was driven by the PLP promoter (PLP-aSyn), hyperphosphorylated aSyn at serine 129 (pSer129) was observed in GCIs. (Kahle et al. 2002). Initial studies using this model did not observe neuronal loss or motor impairments in mice up to 10 months of age; however, subsequent studies found early microglial activation and mild degeneration of nigral dopamine neurons that corresponded to a subtle motor phenotype of reduced hind limb strength. Despite the formation of GCIs, oligodendroglial dysfunction was not observed, supported by the lack of demyelination or oligodendrocyte loss (Stefanova et al. 2005, 2007; Fernagut et al. 2014). Many non-motor and features of dysautonomia have been observed in the PLP-αSyn mouse, such as heart rate variability, impaired respiratory control, bladder dysfunction, REM sleep behavior disorder, (Stefanova et al. 2005; Stemberger et al. 2010; Boudes et al. 2013; Kuzdas et al. 2013; Fernagut et al. 2014; Flabeau et al. 2014; Hartner et al. 2016). Exposure of mitochondrial toxin 3-NP or systemic proteasome inhibition in the PLP-aSyn mouse exacerbated the MSA phenotype and resulted in neuronal loss in the striatum, substantia nigra, inferior olives, pontine nuclei, and cerebellar cortex (Stefanova et al. 2005, 2012). Environmental triggers that induce oxidative stress protein degradation deficits may induce or play a synergistic role in driving the pathology in MSA.

Several tg mouse lines were created using the MBP promoter to overexpress aSyn at varying degrees (MBP-aSyn) (Shults et al. 2005). Fibrillar perinuclear inclusions were formed that were ubiquinated and phosphorylated at Ser129. The most abundant formation of GCI pathology was observed in white matter tracts, the striatum, brainstem, and cerebellum, which corresponded to demyelination and astrogliosis observed throughout white matter tracts, loss of nigral dopamine neurons, reduced striatal TH + fiber density, and loss of striatal neurons. Ultrastructural examination of oligodendrocytes revealed prominent mitochondrial abnormalities, such as enlarged and irregularly shaped organelles, as well as loss of glial cell line-derived neurotrophic (GDNF) (Shults et al. 2005; Ubhi et al. 2010). The decreased levels of GDNF highlight oligodendrocyte dysfunction and point to a mechanism by which oligodendroglial pathology can cause degeneration in neurons. Importantly, GCI burden and subsequent neurodegeneration was dependent on the levels of aSyn expression. The severity of motor phenotype also varied greatly across the different MBP-aSyn lines, with severe tremor, ataxia, seizures and premature death in the highest expressing line (high expressor line 29, MBP29) to mild tremor and variable motor impairment in intermediate and lower expressing lines (Shults et al. 2005). Results from these studies suggest that the level of aSyn expression dictate the degree of neurodegeneration in MSA.

Recently, Mészáros and colleagues investigated line 29 the MBP-aSyn mouse to assess if the overexpression of aSyn under the MBP promoter recapitulates features of the MSA-C variant (Meszaros et al. 2021). Similar to the disease in humans, this model shows GCI formation within cerebellar white matter accompanied by a severe myelin deficit. A significant reduction in MBP and PLP shown by Western Blot was observed at 8 weeks, as well as reductions in myelin lipid content in the pyramidal tract as well as the superior, middle, and inferior cerebellar peduncles. An increase of Olig2 + oligodendrocytes was observed in the cerebellar white matter, while the proportion of PDGFRα + oligodendrocyte precursor cells was slightly decreased (Meszaros et al. 2021). In line with previous studies, these results point to an aSyn mediated dysfunction of oligodendroglia leading to the demyelination observed in MSA, rather than an overt loss of oligodendrocytes (Ettle et al. 2014; May et al. 2014). Accompanying the loss of myelin was an early and sustained increase of IBA1 + cells in the cerebellar white matter beginning at 8 weeks of age in the MBP-aSyn mice, with an 18% loss of Purkinje cells in the cerebellar cortex by 16 weeks. As a consequence of the combined olivopontocerebellar atrophy observed in these mice, gait analyses revealed decreased walking speed, increased stride length and width between hind paws, and less dual diagonal support—akin to the wide-based and unsteady gait often observed in MSA-C patients. Taken together, this model shows features of MSA-C in respect to both pathological findings, as well as a behavioral phenotype that is akin to cerebellar ataxia.

Expression of αSyn under the CNP promoter (CNP-aSyn) resulted in the formation of GCI-like inclusions and neurodegeneration most prominently in the cerebral cortex and spinal cord (Yazawa et al. 2005). CNP-aSyn mice developed an age-related progressive reduction of rotarod performance compared to control mice. Ultrastructural examination of oligodendrocytes from these mice displayed degraded myelin, extensive accumulation of lysosomes, and cytoplasmic myelin fragments, suggesting autophagocytosis. While neuronal cell bodies did not stain positive for human aSyn, positive staining was observed in neurons using antibodies specific for mouse aSyn, indicating the accumulation of endogenous aSyn in neurons of CNP-aSyn mice.

Tg MSA mice have continuous expression of aSyn in oligos, which has been shown to alter oligodendroglial maturation (Ettle et al. 2014; May et al. 2014). As such, forced expression of aSyn throughout development could result in disrupted brain circuitry, and thus deficits observed in these tg models may not entirely represent the disease course in MSA. Recently, Tanji and colleagues developed an adult-onset aSyn overexpression model of MSA using a Cre-loxP system in mice to induce aSyn expression in oligodendroglia at chosen time (Tanji et al. 2019). To generate this model, mice containing a chicken β-actin gene and a promoter (CAG promoter) followed by a loxP-flanked (flox) stop cassette-controlled aSyn gene were mated with mice that contained the Cre/estrogen receptor (ER) specifically expressed under the PLP promoter. As such, injection of tamoxifen induced overexpression of aSyn specifically in oligodendrocytes that was phosphorylated at Ser129 and resistant to proteinase K digestion. aSyn expression gradually spread throughout the CNS, mainly in white matter of the brainstem and cerebellum, but also in grey and white matter of the spinal cord. As GCI formation, neuronal cytoplasmic and neuronal nuclear inclusions were observed in the putamen and brainstem. Interestingly, 30% of the male mice exhibited sudden death following tamoxifen injection. Beginning 50 weeks following tamoxifen injection, behavioral abnormalities began to appear, as well as increased Iba1 and GFAP staining intensity. No neuronal loss was observed, however KB staining showed relatively weaker myelin staining in the spinal cord (Tanji et al. 2019). The tamoxifen inducible MSA mouse avoids the pitfall of sustained aSyn observed in tg MSA mice by inducing expression in adult mice, however, the GCIs formed in both the tg and inducible MSA mice are not specific to those regions affected in MSA. Both the tg MSA mice and the inducible adult-onset MSA mice overexpress in all oligodendroglia throughout the CNS, not just those regions affected by MSA.

### Viral vector-mediated models

To allow for temporal control and region-specific overexpression of aSyn, our group and others have turned to AAVs to model MSA in rodents and NHPs. Bassil and colleagues utilized AAV1/2 to overexpress aSyn or GFP specifically in oligodendrocytes under the MBP promoter (Bassil et al. 2017). Targeting expression to the striatum, ~ 80% oligodendrocyte-specific transduction was observed in rats, which dropped to ~ 60% in rhesus macaques. Prominent aSyn accumulation was observed, which was pS129 + and resistant to proteinase K digestion. Progressive L-dopa unresponsive behavioral deficits was reported in rats overexpressing aSyn, which was accompanied by neurodegeneration beginning at 3 months and progressing at 6 months post-injection, with a 23% loss of NeuN + neurons in the striatum and a 66% loss of TH + nigral neurons (Bassil et al. 2017).

By taking advantage of AAV engineering techniques such as  capsid shuffling and directed evolution, our colleagues were able to design an AAV capsid which displayed specific tropism towards oligodendroglia and this vector was called Olig001 In vitro binding assays showed that Olig001 transduced oligodendroglia 9 times more than AAV8 serotype in mixed cell cultures (Powell et al. 2016). Following striatal injection of Olig001 expressing aSyn or GFP under the constitutive CBh promoter, we found > 95% oligodendroglial-specific tropism in rats and 90–94% in rhesus macaques (Mandel et al. 2017). In rats, neurodegeneration corresponded with aSyn expression levels, where greater levels of demyelination and neuronal loss in both the striatum and SN were observed when using a tenfold higher titer of Olig001 (Marmion et al. 2021). In both species, widespread aSyn-rich GCIs were present, in which aSyn was phosphorylated at serine 129 and tyrosine 39. These aggregates were resistant to proteinase K, and were Thioflavin S positive, demonstrating the formation of GCIs similar to those observed in MSA. Titer-dependent degeneration was observed with a 14.7% loss of striatal NeuN + neurons and 21.7% loss of TH + nigral neurons in low-titer Olig001-aSyn injected rats (2.4 × 10^11^ vg/ml), which increased to a 23.8% and 35.9% loss of neurons in the striatum and SN of high-titer Olig001-aSyn injected rats (3.2 × 10^12^ vg/ml), respectively (Marmion et al. 2021). Progressive degeneration was observed in the Olig001-aSyn NHP model of MSA, with demyelination observed but no cell loss after 3 months (Mandel et al. 2017). However, there was  a ~ 44% loss of NeuN + neurons in the putamen and a 11% loss of TH + nigral neurons was reported 6 months following injection of Olig001-aSyn, recapitulating the progressive nature of MSA. Robust increases in neuroinflammatory markers, such as HLA-DR, GFAP, and CD3, were observed in aSyn expressing monkeys (Marmion et al. 2021).

To assess the immune response, we injected the Olig001 vector expressing either GFP or aSyn into the striatum of mice (Williams et al. 2020). Using both histology and flow cytometry, we observed a marked increase of MHCII expression on microglia, as well as an infiltration of pro-inflammatory monocytes into the CNS of animals injected with Olig001-aSyn, which was not observed in GFP injected controls. We also observed robust infiltration of CD4 and CD8 + T cells into the CNS and antigen-experienced CD4 and CD8 + T cells in the draining cervical lymph nodes following oligodendroglial expression of aSyn. Cytokine analyses revealed a significant increase in all Th1, 2, 17, and Treg associated cytokines IFN-γ, IL-4, IL-17a, and IL-10, respectively. To investigate the protective effect of reducing T cells, *Tcrb*^−/−^ (CD4 and CD8 T cell knockout), or *Cd4*^−/−^ (CD4 T cell knockout) mice were injected with Olig001-aSyn and the CNS myeloid response was measured using histology and flow cytometry 4 weeks post-transduction. *Tcrb*^−/−^ and *Cd4*^−/−^ Olig001-SYN treated mice displayed a robust decrease in MHCII expression in both microglia and monocytes. Furthermore, *Tcrb*^−/−^ and *Cd4*^−/−^ mice injected with Olig001-aSyn showed reduced neurodegeneration, as no loss of myelin was observed in these mice compared to WT mice (Williams et al. 2020). Histological analysis of post-mortem brain tissue from MSA patients revealed significant increases of HLA-DR + microglia, CD3 + , CD4 + , and CD8 + T cells in the putamen and SN compared to control subjects, confirming our findings from the Olig001 model (Williams et al. 2020). These results suggest that T-cell priming and infiltration into the CNS are key mechanisms of disease pathogenesis in MSA, and therapeutics targeting T cells may be disease modifying.

The use of viral vector technology also allows systemic targeting approaches for experimental or therapeutic purposes. We tested different rAAV viral vector serotypes, including rAAV2/7, rAAV2/8 and rAAV2/9, with different oligodendroglial specific promoters (Peelaerts et al. 2021). By combining rAAV2/9 with the myelin-associated glycoprotein (MAG) promoter expression specificity reached over 95% in oligodendrocytes and it was possible to inject into the lateral ventricles and achieve widespread distribution of the viral vector in mouse CNS. Transgene expression was detected specifically in oligodendrocytes of the white matter spinocerebellar and spino-olivary tracts and the midbrain, including the pons and the cerebellar peduncles, which are areas that are typically affected in MSA. Future experiments will determine of such a model could be used for MSA modelling after injection of viral vector or in combination with seeding of MSA-specific fibrils.

### Fibrilar seeding models of MSA

Finally, a model of MSA has been created via injecting aggregated aSyn fibrils directly into rodent brain. Because of the prion features of aggregated aSyn, fragmented aSyn fibrils can amplify and template in a strain-dependent manner and propagate pathology in vivo (Brundin and Melki 2017). Although the seeding capacities of fibrillar aggregates of aSyn have been useful for the development several PD models (Chung et al. 2019), it has been surprisingly difficult to establish seeding models that recapitulate features of MSA. Some studies have reported glial pathology in rodent brain, with inclusion formation in oligodendrocytes after injection of aSyn fibril injection (Peelaerts et al. 2015; Peng et al. 2018; Uemura et al. 2019). However, injection of MSA-derived fibrils or MSA strains, from patient brain into wild type, transgenic or viral vector models causes aggressive pathology with neuronal inclusions, neuroinflammation and motor behavioral deficits but without the typical MSA-like features such as GCIs, demyelination and brain atrophy (Peng et al. 2018; Holec and Woerman 2020; Van der Perren et al. 2020). The reasons for this are not fully understood, but a possible hypothesis is that an incomplete cellular environment in these models does not allow the propagation of MSA-like pathology. Peng and colleagues injected oligodendroglia-derived seeded fibrils in transgenic mice expression aSyn in oligodendrocytes where they observed progressive oligodendrogliopathy (Peng et al. 2018). Similarly, injection of aSyn ribbons or fibrils into MSA transgenic mice resulted in a significant behavioral changes, brain atrophy with demyelination and neuronal loss but also distinct MSA-like phenotypes (Torre Murazabal et al. 2020). It therefore seems that an interaction between host environment and aSyn assemblies dictate the conformational nature and the structural variation of a complex phenotype in MSA. Future studies will have to determine how reliable seeding models could be established, which will provide further insights into the mechanisms of disease progression in MSA.

## Conclusion

**Fig. 1:**
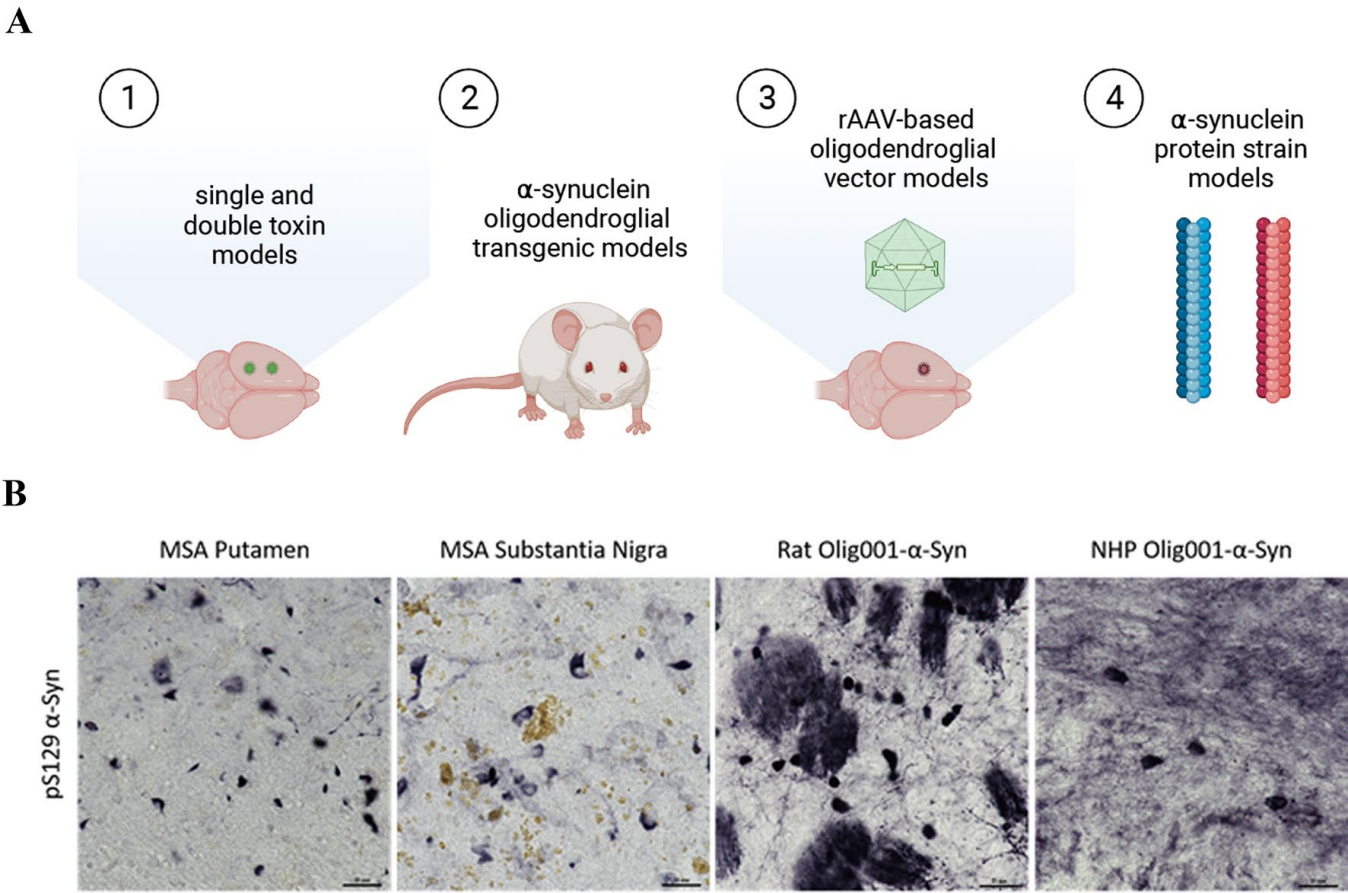
Summary of animal models of MSA and recapitulation of MSA pathology. Animals have been utilized to model MSA in vivo using different approaches, such as 1. Toxin based models in rodents and NHPs, 2. Transgenic mouse models overexpressing aSyn in oligodendroglial-specific promoters, 3. Virial vector-mediated aSyn overexpression models in rodents and NHPs, 4. aSyn protein strain models (**A**). Pathological phosphorylation of aSyn at Serine residue 129 is observed in post-mortem MSA tissue in both the putamen and SN, which is recapitulated in the striatum of rats and nonhuman primates injected with oligodendroglial-specific AAV capsid Olig001-aSyn (**B**). Scale bars = 25 μm

The sequence of events that initiates and drives the pathogenesis of MSA is currently not fully understood. While post-mortem tissue from MSA patients provide a snapshot of end-stage disease, recent developments with cell and animal models of MSA (detailed  in Table 1, Fig. 1) have better recapitulated critical aspects of the human pathology and molecular dynamics underlying the degenerative process, which have provided some insight into our understanding of the pathogenesis of MSA. The relocation of p25α from the myelin sheath to the soma of oligodendroglia to form GCIs seems to precede and initiate the aggregation of aSyn assemblies. The source of aSyn in MSA is unclear- as either aberrant expression of aSyn in oligodendroglia or the uptake of secreted aSyn from neurons may occur in MSA to form GCIs. Regardless, the formation of proteinaceous GCIs leads to a number of secondary events involving the dysfunction of oligodendrocytes, such as the disruption of myelin homeostasis, reduced trophic support to axons and neurons, and increased neuroinflammation. As such, MSA is currently viewed as a primary synucleinopathy with disruptions in the oligo-myelin-axon-neuron complex and secondary neurodegeneration (Wenning et al. 2008).

**Table 1:** Overview of animal models of multiple system atrophy with key phenotypes

Model class	Model Type	Behavioral deficits	Oligodendroglial dysfunction (demyelination)	Neuronal loss			Gliosis and inflammation	Synuclein pathology	References
				SND	OPCA	SC			
Toxin	6-OHDA and QA	** + + + **	−	** + + + **	−	−	+ + (GFAP)	NA	Wenning et al (1996)
	6-OHDA and QA	** + + + **	−	** + + + **	−	−	+ + (GFAP)	NA	Scherfler et al. (2000),
	6-OHDA and QA	** + + + **	−	** + + + **	−	−	NA	NA	Stefanova et al. (2004)
	6-OHDA and QA	** + + + **	−	** + + + **	−	−	NA	NA	Kollensperger et al. (2007)
	6-OHDA and QA	** + + + **	−	** + + + **	−	−	+ + (GFAP)	NA	Ghorayeb et al. (2001)
	3NP, MPTP	** + + + **	−	** + + + **	−	−	+ + (GFAP)	NA	Ghorayeb et al. (2002a, b)
	3NP, MPTP	** + + + **	−	** + + + **	−	−	+ + (GFAP)	NA	Stefanova et al. (2003a, b)
	3NP, MPTP *(non-human primates)*	** + + + **	−	** + + + **	−	−	NA	NA	Ghorayeb et al. (2002a, b)
Transgenic	PLP-aSyn	** + **(dysautonomia)	−	** + **	+ *	** + **	** + **	** + + **	Kahle et al. (2002)
	MBP-aSyn	** + **	** + + **(↓ GDNF)	** + + **	** + **	NA	** + **	** + + **	Shults et al. 2005
	CNPase-aSyn	** + **	** + **	** + + **	NA	** + **	** + **	** + + **	Yazawa et al. (2005)
	CAG-loxP-aSyn-loxP x PLP-Cre	** + **	−	−	−	−	** + **	** + + **	Tanji et al. (2019)
Viral vector	rAAV2/1 MBP-aSyn *(rat and non-human primate)*	+ + (l-dopa unresponsive)	NA	+	NA	NA	NA	+ +	Bassil et al. 2017
	Olig001-CBh-aSyn *(rat and non-human primate)*	−	+	−	NA	NA	+ +	+ +	Mandel et al. (2017)
	Olig001-CBh-aSyn *(rat and non-human primate)*	−	+	+	NA	NA	+ + + (GFAP, T-Cells)	+ +	Marmion et al. (2021)

*NA* Not assessed,—no effect, + weak effect, +  + moderate effect, +  +  + strong effect, * additional hit with neurotoxin.. 6-OHDA: 6-hydroxy-dopamine, *QA* quinolic acid, *3NP* 3-nitropropionic acid, *MPTP* 1-methyl-4-phenylpyridinium ion, *CAG* CMV enhancer, chicken beta-actin, *PLP* proteolipid protein, *MBP* myelin basic protein, *CNPase* 2’, 3’-cyclic-nucleotide 3’phosphodiesterase

To date, there are no causative or disease-modifying therapies available for MSA, and symptomatic treatments are limited. Initial responsiveness to levodopa has been reported to be beneficial in 83% of MSA-P patients (Kollensperger et al. 2010), which declines to 31% effectiveness after a period of 3.5 years (Wenning et al. 2013). In fact, the diminishing effectiveness of l-dopa often assists in the diagnosis of MSA. Other symptomatic treatment relies on the use of midodrine and droxidopa for orthostatic hypotension, anticholinergic agents for urinary tract dysfunction, laxative therapy for constipation, continuous positive air pressure and tracheotomy for breathing disorders, and botulinum toxin injections for dystonia (Perez-Lloret et al. 2015). A number of potential disease-modifying therapies for MSA are currently being tested in animal models of the disease ((Heras-Garvin and Stefanova 2020).

Currently, there are four clinical trials underway targeting aSyn in MSA (https://clinicaltrials.gov/ct2/show/NCT04165486, https://clinicaltrials.gov/ct2/show/NCT02270489, https://clinicaltrials.gov/ct2/show/NCT03100149), as well as 1 clinical trial examining the effects of GDNF gene delivery (https://clinicaltrials.gov/ct2/show/NCT04680065) in this population. In the realm of aSyn degradation enhancers, a phase II, double-blind, futility trial is being conducted with Rapamycin to target aSyn clearance in MSA patients (Palma et al. 2019). Results from a phase I study suggest good immunogenicity, safety and tolerability for active immunization using two vaccines, PD01A and PD03 targeting aSyn aggregation in MSA patients (Meissner et al. 2020). Two trials are underway targeting aSyn aggregation. Anle138b, an oral small compound targeting aSyn aggregation, has shown promise in PLP-aSyn MSA mice and a phase I trial in MSA patients has recently been announced (Heras-Garvin et al. 2019) https://www.modag.net/images/pressrelease_modag_series_a.pdf). Additionally, a phase I trial with healthy volunteers is currently ongoing with small molecule iron chaperone PBT434, targeting iron-mediated aSyn accumulation, where preliminary results showed proportional pharmacokinetics up to 300 mg and good tolerance (Stamler et al. 2019). Targeting neuroinflammation, a phase IIa trial in MSA patients with myeloperoxidase inhibitor Verdiperstat has shown improvement on clinical scores and reduced neuroinflammation by PET imaging (NCT02388295). A phase III study with Verdiperstat in MSA is planned (https://clinicaltrials.gov/ct2/show/NCT04616456). Intrathecal administration of autologous mesenchymal stem cells has shown neuroprotective and immunomodulatory effects in animal models (Park et al. 2011; Stemberger et al. 2011). Studies in MSA patients have shown improvement on clinical scores and the safety profile examined through phase I/II study (Lee et al. 2008, 2012; Singer et al. 2019). However, a large, randomized, placebo-controlled study will be necessary before this approach is translated into the clinic. Based on the previously discussed findings on disruptions of CoQ10 levels in Asian patients with MSA-C, supplementation with CoQ10 is currently being investigated in a randomized, double-blind, placebo-controlled, multicenter phase II trial (UMIN000031771).

In summary, several factors seem to be associated with the pathogenesis of MSA and are potential therapeutic targets for disease modification. Continued collaboration between clinical and pre-clinical researchers is necessary to bridge the knowledge gaps surrounding the origin, cause, and multifaceted nature of MSA. Moreover, further work is needed towards the identification of early biomarkers for MSA, not only for better diagnostic aspects, but to implement future disease modifying therapies at an early stage.
